# The Antiangiogenic Compound Aeroplysinin-1 Induces Apoptosis in Endothelial Cells by Activating the Mitochondrial Pathway

**DOI:** 10.3390/md10092033

**Published:** 2012-09-18

**Authors:** Beatriz Martínez-Poveda, Salvador Rodríguez-Nieto, Melissa García-Caballero, Miguel-Ángel Medina, Ana R. Quesada

**Affiliations:** 1 Department of Molecular Biology and Biochemistry, Faculty of Sciences, University of Málaga, Málaga E-29071, Spain; Email: bmpoveda@gmail.com (B.M.-P.); melissa@uma.es (M.G.-C.); medina@uma.es (M.-A.M.); 2 Genes and Cancer Group, Cancer Epigenetics and Biology Program (PEBC-IDIBELL), Barcelona E-08908, Spain; Email: srodriguezn@idibell.cat; 3 Centre for Biomedical Network Research on Rare Diseases (CIBERER), Málaga E-29071, Spain

**Keywords:** aeroplysinin-1, angiogenesis, apoptosis, caspases

## Abstract

Aeroplysinin-1 is a brominated metabolite extracted from the marine sponge *Aplysina aerophoba* that has been previously characterized by our group as a potent antiangiogenic compound *in vitro* and *in vivo*. In this work, we provide evidence of a selective induction of apoptosis by aeroplysinin-1 in endothelial cells. Studies on the nuclear morphology of treated cells revealed that aeroplysinin-1 induces chromatin condensation and nuclear fragmentation, and it increases the percentage of cells with sub-diploid DNA content in endothelial, but not in HCT-116, human colon carcinoma and HT-1080 human fibrosarcoma cells. Treatment of endothelial cells with aeroplysinin-1 induces activation of caspases-2, -3, -8 and -9, as well as the cleavage of apoptotic substrates, such as poly (ADP-ribose) polymerase and lamin-A in a caspase-dependent mechanism. Our data indicate a relevant role of the mitochondria in the apoptogenic activity of this compound. The observation that aeroplysinin-1 prevents the phosphorylation of Bad relates to the mitochondria-mediated induction of apoptosis by this compound.

## 1. Introduction

Angiogenesis, the generation process of new capillaries from a pre-existing vascular bed, is a highly regulated mechanism of vascularization, which in adults is restricted to processes such as the female reproductive cycle and wound healing. Nevertheless, abnormal angiogenesis has been related to a number of pathological processes, such as tumor growth, metastasis, diabetic retinopathy, age-related macular degeneration, psoriasis and arthritis, among others [1]. For this reason, angiogenesis inhibition has attracted extensive attention in the field of pharmacological research, as well as becoming an emergent approach for the treatment of cancer and other angiogenesis-related diseases [2]. Although clinical experience indicates some limitations of these therapies, the increasing number of antiangiogenic agents that have been approved for the treatment of cancer and blindness encourages expectations on their therapeutic potential [3]. 

**Figure 1:**
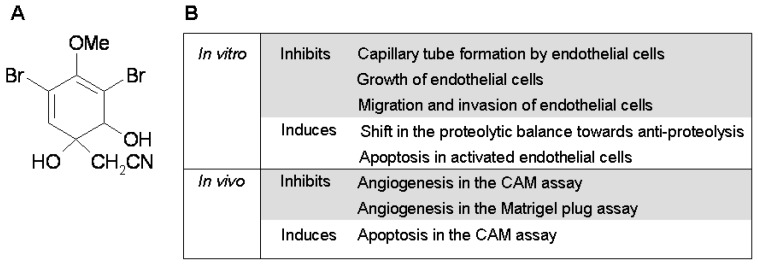
(**A**) Chemical structure of aeroplysinin-1; (**B**) Summary of its anti-angiogenic properties.

Aeroplysinin-1 is a naturally occurring brominated tyrosine metabolite extracted from the marine sponge *Aplisina aerophoba*, where it seems to play a defensive role [4] (Figure 1A). In the course of a blind high-throughput screening for new potential inhibitors of angiogenesis obtained from marine organisms, our group selected aeroplysinin-1 and characterized it as a potent antiangiogenic compound *in vitro* and *in vivo *[5]. Aeroplysinin-1 was shown to inhibit angiogenesis in the chick chorioallantoic membrane and in subcutaneous Matrigel implants in mice, and to interfere with key steps of angiogenesis in endothelial cells, including proliferation, migration, capillary tube formation and the ability to invade and remodel the extracellular matrix. In a previous work, we demonstrated that aeroplysinin-1 could induce apoptosis in endothelial cells, although the mechanisms leading to apoptosis remained elusive (Figure 1B). 

Likewise, it has been previously reported that a number of endogenous and exogenous angiogenesis inhibitors induce endothelial cell apoptosis, including thrombospondin-1 [6], pigment epithelium-derived factor [7], adiponectin [8], neovastat [9] and IB05204 [10], among many others. It has been suggested that endothelial cell apoptosis induced by a variety of mechanisms might be responsible for their angiogenesis-inhibitory activity. During apoptosis, extrinsic or intrinsic signals activate caspases, which in turn induce DNA fragmentation, DNA budding, and the chromatin condensation characteristic of programmed cell death (reviewed in [11]).

Here, we further characterize the pro-apoptotic activity of aeroplysinin-1 in endothelial cells, analyzing different features of the apoptotic process, such as activation of caspases, alteration of the mitochondria, specific cleavage of death substrates and nuclear fragmentation. Our results suggest that the apoptosis-inducing mechanism of aeroplysinin-1 is endothelial cell-specific and dependent on the apoptogenic mitochondrial pathway through activation of the BH3-only pro-apoptotic protein Bad, cyrochrome *c* release and activation of caspases 2, 3, 8 and 9.

## 2. Results and Discussion

### 2.1. Aeroplysinin-1 Inhibits the Growth of Endothelial and Tumor Cells

**Figure 2:**
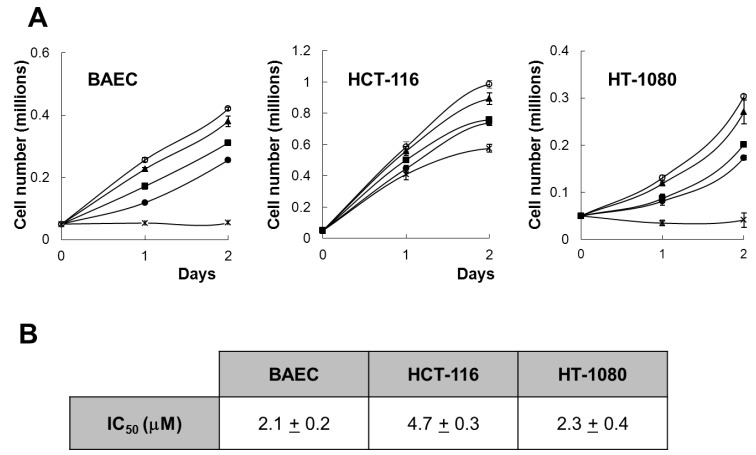
Effect of aeroplysinin-1 on endothelial and tumor cell growth. (**A**) Cell counts of BAEC, HCT-116 and HT1080 grown either in the absence or the presence of 1 µM, 3 µM, 5 µM or 10 µM aeroplysinin-1 (respectively marked as o, ▲, ■, ● and x). Values are means ± SD of quadruplicate samples. (**B**) Half-maximal inhibitory concentration (IC50) values were calculated from MTT dose-response curves as the concentration of aeroplysinin-1 yielding 50% of control cell survival. They are expressed as means ± SD of three independent experiments with quadruplicate samples each.

The effect of aeroplysinin-1 on endothelial and tumor cell growth was investigated by cell count and by MTT assay. As shown in Figure 2A, aeroplysinin-1 inhibited, in a concentration-dependent manner, the growth of BAEC, HCT-116 and HT-1080, with a complete inhibition of BAEC and HCT-116 cell proliferation, exerted at a concentration of 10 µM. This is in agreement with the antiproliferative effect of aeroplysinin-1 on endothelial cells, previously described by us [5]. Data obtained with the colorimetric MTT assay (Figure 2B) show that aeroplysinin-1 is not a specific inhibitor of endothelial cell growth, as the half-maximal inhibitory concentration (IC50) value of this effect on endothelial cells (BAECs) is in the same range as those obtained with tumor cells (HCT-116 and HT-1080). 

### 2.2. Aeroplysinin-1 Induces Apoptosis in Endothelial Cells

**Figure 3:**
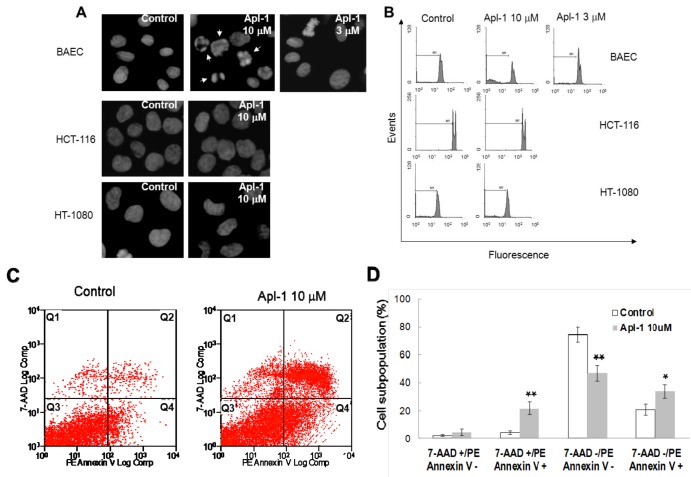
Aeroplysinin-1 potentiates apoptosis in endothelial cells. (**A**) Nuclear morphology of endothelial and tumor cells after treatment with aeroplysinin-1, assessed under a fluorescence microscope. (**B**) Cell cycle distribution of endothelial and tumor cells after treatment with aeroplysinin-1, analyzed by FACS. M1 indicates the subG1 population. (**C**) Determination of endothelial cell (BAEC) apoptosis by flow cytometry analysis after PE-Annexin V and 7-aminoactinomycin D (7AAD) staining. (**D**) 7AAD−/PE-Annexin V−, 7AAD−/PE-Annexin V+, 7AAD+/PE-Annexin V+ and 7AAD+/PE-Annexin V− populations, corresponding to viable (Q3), early apoptotic (Q4), late apoptotic (Q2) and necrotic (Q1) cells respectively, were evaluated as in (**C**). Values are expressed as means ± SD of three independent experiments. * *p* < 0.05; ** *p* < 0.005 *versus* control.

As a first approach to study the effect of aeroplysinin-1 in endothelial and tumor cells, the nuclear morphology of BAE, HT1080 and HCT116 cells was analyzed by Hoechst staining after 14 h treatment with this compound. As shown in Figure 3A, treatment with 10 μM aeroplysinin-1 induced chromatin condensation and nuclear fragmentation in endothelial (BAE) cells, but not in colon carcinoma (HCT-116) or fibrosarcoma (HT-1080) cells. Nuclei of BAE cells treated with 3 μM aeroplysinin-1 did not show morphological changes compared to nuclei of non-treated cells, showing a dose dependence of this effect that is in agreement with that observed in the cell growth assay, where 10 μM aeroplysinin-1 was required to completely inhibit proliferation of BAEC. To confirm these results, the cell cycle distribution of propidium iodide-stained cells was analyzed by flow-cytometric analysis. Figure 3B shows that a significant increase (6-fold) in the sub-G1 population was observed in BAE cells treated with 10 μM aeroplysinin-1 when compared to untreated cells. Nevertheless, no significant increases of sub-diploid population were observed in either endothelial cells treated with 3 μM aeroplysinin-1, or in HCT-116 or HT-1080 cells treated with 10 μM aeroplysinin-1 (Figure 3B). These results suggested that aeroplysinin-1 might be a selective apoptosis trigger in endothelial cells. Endothelial cell apoptosis induced by aeroplysinin-1 was also confirmed by flow cytometry analysis after PE-Annexin V and 7-aminoactinomycin D (7AAD) staining (Figure 3C,D), showing that 10 µM aeroplysinin-1 induced a significant increase in the percentage of cells in both early and late apoptotic cell subpopulations. 

### 2.3. Aeroplysinin-1 Induces Activation of Caspases and Cleavage of PARP and Lamin-A in Endothelial Cells

**Figure 4:**
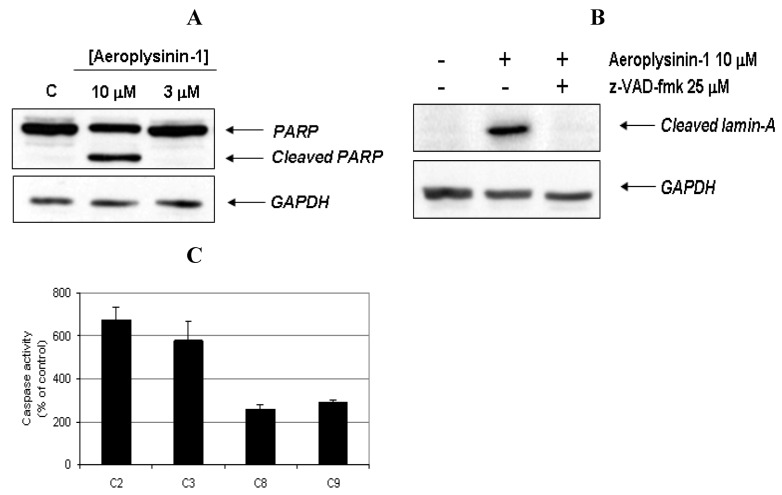
Activation of caspases and cleavage of apoptotic substrates induced by aeroplysinin-1. (**A**) Western-blot detection of PARP cleavage in aeroplysinin-1-treated BAE cells. Glyceraldehyde 3-phosphate dehydrogenase (GAPDH) was used as loading control. (**B**) Western-blot detection of cleaved lamin-A in aeroplysinin-1-treated BAE cells with or without pre-incubation of cells with the pan-caspase inhibitor z-VAD 25 μM. (**C**) Effect of 10 µM aeroplysinin-1 in BAE cells caspase-2, -3, -8 and -9 activation. Activity values from treated cells are expressed as percentage of untreated (control) cells. Bars represent the standard deviation from duplicated samples in the same assay. Similar results were obtained in three independent experiments.

In order to provide biochemical evidence for the induction of apoptosis in BAE cells treated with aeroplysinin-1, the cleavage of poly (ADP-ribose) polymerase (PARP) and lamin-A, was studied. PARP cleavage by activated caspase-3 is a key event in the process of apoptosis and is used as an early marker for apoptosis induction [12]. As shown in Figure 4A, PARP was cleaved from 116 kDa intact form into 85 kDa fragment after BAE cell treatment with 10 μM of aeroplysinin-1. Lamin-A is a structural protein belonging to the intermediate filament family that, together with other lamin species, constitutes the scaffolding of the nuclear envelope. During apoptosis, lamin A is cleaved at Asp230 by caspase-6 [13] causing the characteristic collapse of the nucleus observed during apoptosis. Treatment of BAEC with 10 µM aeroplysinin-1 caused lamin A cleavage, generating a fragment of 28 kDa. Based on those results, we reasoned whether aeroplysinin-1 induced apoptosis could be mediated by caspases, and used a pharmacological inhibitor of caspases to gain insight into this question. As shown in Figure 4B, the addition of 25 µM of the pan-caspase inhibitor *N*-benzyloxycabonyl-Val-Ala-Asp-fluoromethylketone (ZVAD) inhibited lamin A cleavage, indicating that aeroplysinin-1 induces apoptosis in BAE cells through a caspase-dependent pathway.

To further confirm that aeroplysinin-1 induced cell death is a caspase-dependent process, the activities of different caspases were measured. Fluorometric assays using specific substrates showed that 14 h treatment of BAE cells with 10 µM aeroplysinin-1 induced a 7 fold increase of caspase-2 activity, a 6 fold increase of caspase-3 and a 3 fold increase of caspases-8 and -9, as compared to the activity of untreated cells (Figure 4C). Inhibition of caspase-9 after the addition of the specific caspase-9 inhibitor LEHD-fmk significantly abrogated aeroplysinin-1-induced activation of caspase-2 and caspase-3 (results not shown), indicating the relevant role of caspase-9 as an upstream caspase in the mitochondria-mediated apoptosis pathway. 

### 2.4. Aeroplysinin-1 Induces Cytochrome c Release in Endothelial Cells

**Figure 5:**
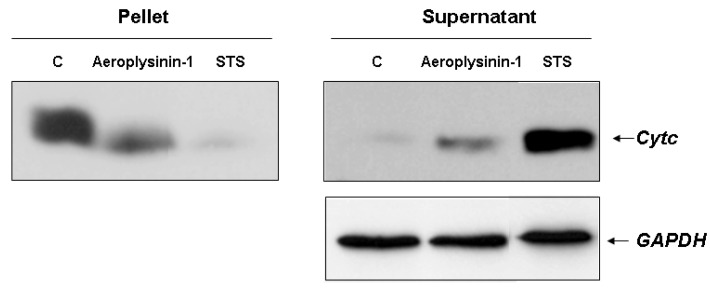
Detection of cytochrome *c* release in 10 µM aeroplysinin-1-treated BAE cells. Treatment of BAEC with staurosporine (STS) 2 µM was used as internal control of mitochondrial cytochrome *c* release. Representative blots from two different experiments are shown.

Our data, suggesting a relevant role of caspase-9 activation in the aeroplysinin-1 apoptogenic activity, prompted us to investigate alterations in the physiology of the mitochondria caused by this drug. The intrinsic or mitochondrial apoptotic pathway is characterized by a permeabilization of the outer mitochondrial membrane induced by the pro-apoptotic stimuli, leading to the release of cytochrome *c* from the intermembrane space into the cytosol. Cytochrome *c* is a crucial component of the mitochondrial pathway of apoptosis, since by binding to the cytosolic apoptotic protease-activating factor 1 (Apaf-1), it contributes to the apoptosome assembly, which acts as an activation platform for procaspase-9 [14]. This feature was studied in digitonin-permeabilized BAE cells treated with 10 µM aeroplysinin-1 as described in *Experimental Section. *Treatment with aeroplysinin-1 elicited the redistribution of cytochrome *c* from mitochondria to cytosol, although the release seemed to be incomplete, as shown by the presence of cytochrome *c* in the pellet (Figure 5).

### 2.5. Aeroplysinin-1 Inhibits BAD Phosphorylation in Endothelial Cells

**Figure 6:**
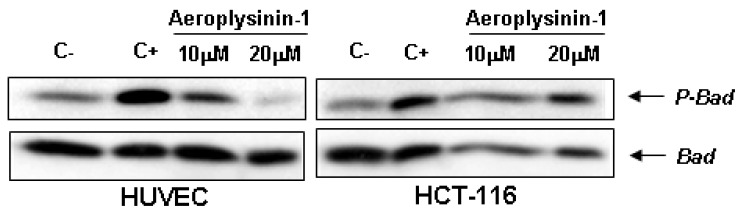
Effects of aeroplysinin-1 on Bad phosphorylation in human umbilical vein endothelial cells (HUVEC) and human HCT-116 colon carcinoma cells. Negative control (C−) corresponds to starved but not induced cells; positive control (C+) corresponds to starved and serum-induced cells. Identical results were obtained in two independent experiments.

The BH3-only pro-apoptotic protein Bad is regulated by phosphorylation-dephosphorylation in response to extracellular stimuli. The phosphorylation status of Bad determines its pro-apoptotic activity at the mitochondrial level [15], facilitating its depolarization by interacting and restraining the anti-apoptotic protein Bcl-XL. Therefore, we decided to study the effect of aeroplysinin-1 on the phosphorylation status of Bad in endothelial cells by serum deprivation and re-stimulation, with or without addition of aeroplysinin-1. As shown in Figure 6, aeroplysinin-1 prevented phosphorylation of Bad in HUVE cells in a dose-dependent manner, but not in HCT-116 cells, reinforcing the previously shown data regarding the endothelial selectivity of aeroplysinin-1 apoptogenic activity (Figure 3 and Figure 6). 

### 2.6. Global Discussion

Aeroplysinin-1 has been previously described by us as a potent antiangiogenic compound, showing inhibitory effects *in vitro* and *in vivo*. In addition, it was suggested to trigger apoptosis in endothelial cells, although no results characterizing this process have been provided to date [5]. The present work provides evidence showing that aeroplysinin-1 effectively induces apoptosis in endothelial cells via the apoptogenic mitochondrial pathway, with inhibition of Bad phosphorylation, cytochrome *c* release from mitochondria and activation of caspases.

Our previous results indicated that aeroplysinin-1 inhibits the growth of highly proliferant BAE cells, and it exhibits a significantly lower activity on low-proliferant cells [5]. Results shown in this paper confirm the antiproliferative activity of this compound on endothelial and tumor cells. Moreover, our previous suggestion that aeroplysinin-1 could induce cell death by activation of apoptosis is now confirmed by new experimental data, revealing that incubation with this compound induces several changes on endothelial cells which are indicative of apoptosis, including chromatin condensation and nuclear fragmentation, an increase in the percentage of cells with sub-diploid DNA content, and an increase in the Annexin V+ subpopulations. Interestingly, the same doses of aeroplysinin-1 did not cause any observable effect in the human HCT-116 and HT-1080 tumor cell lines, which suggests a selective induction of apoptosis in endothelial cells. 

Activation of caspases is considered to be the prerequisite to define apoptotic cell death [16]. Here, we demonstrate that aeroplysinin-1 induces activation of caspase-2, -3, -8 and -9 in endothelial cells, in parallel with the cleavage of hallmark apoptotic substrates, such as PARP and lamin-A. Selective inhibition of caspase-9 by LEHD-fmk abrogated activation of caspases-2 and -3 by aeroplysinin-1, suggesting it to be dependent on caspase-9 activation upstream of caspase-2 and -3. Caspase-2 is a putative initiator caspase characterized by the presence of a caspase activation recruitment domain (CARD) and structurally related to the initiator caspase-9. The precise mechanism of caspase-2 activation during apoptosis is the subject of intense research [17] and seems to be dependent on the nature of the stimulus. The activation of caspase-2 in response to genotoxic stress has been shown to occur within a PIDDosome complex [18] and this process is dependent on p53 function [19]. The role of caspase-2 remains controversial as to whether it is either an essential initiator caspase acting upstream of mitochondria [20], or an executioner caspase activated downstream of mitochondria [21]. Our results suggest that aeroplysinin-1-induced caspase-2 activation in endothelial cells depends on caspase-9 activity and hence, it occurs downstream of mitochondria. 

The linked observations of the presence of caspase-9 activity and the induction of cytochrome *c* release in aeroplysinin-1-treated endothelial cells suggest an implication of mitochondria in this model. Mitochondria plays a central role in the transmission of the apoptotic cascade [14]. Depolarization of the outer mitochondrial membrane causes the release of different pro-apoptotic proteins from the mitochondrial intermembrane space to the cytosol, such as cytochrome *c*, which interacts with the adaptor protein Apaf-1 and constitutes the platform for the activation of caspase-9 and downstream executioner caspases [22].

The Bcl-2-related BH3-only pro-apoptotic protein Bad is an upstream sensor of cellular damage that is activated through phosphorylation and dephosphorylation processes in response to extracellular stimuli [23]. Dephosphorylated Bad heterodimerises with Bcl-XL in the outer mitochondrial membrane, leading to changes in mitochondria permeability and promoting apoptosis [15]. Phosphorylation of Bad in serine residues results in its association with the scaffold protein 14-3-3, which sequester Bad in the cytosol, preventing its pro-apoptotic effect. Although Bad can be phosphorylated in some serine residues, Ser112 and Ser136 are the residues implicated in the association of this protein with 14-3-3 [24]. In fact, the phosphorylation state of Bad has been proposed to decrease the vulnerability of the mitochondria to release cytochrome *c* and induce apoptosis in response to death signals [25]. 

**Figure 7:**
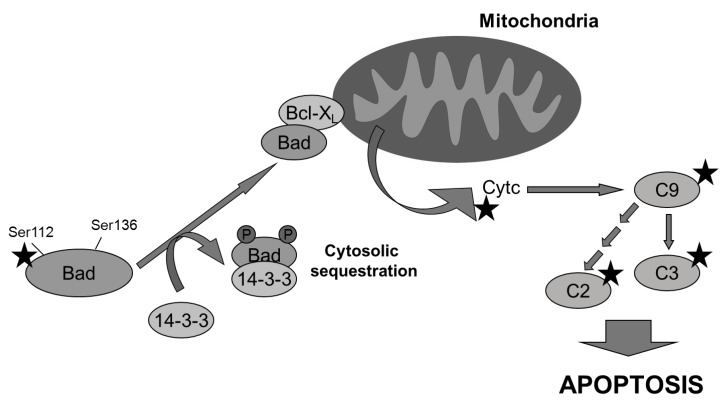
Multiple molecular targets (indicated with a star) are involved in the pro-apoptotic effect of aeroplysinin-1 observed in endothelial cells. Bad is dephosphorylated and it translocates from cytosol to mitochondria, where it dimerizes with Bcl-XL and promotes the release of cytochrome *c*, activating caspase-9 (C9) and other downstream caspases.

Little is known about aeroplysinin-1 and how it can selectively target endothelial cells. Several reports provide evidence concerning the inhibitory effect of aeroplysinin-1 on EGF receptor *in vitro* and *in vivo* [26,27], although this effect has been questioned by others [28]. No data is available about the inhibitory capacity of aeroplysinin-1 on key steps of the signal transduction pathways controlling angiogenesis. Taken together, our results led us to suggest a mechanism by which aeroplysinin-1, through inhibition of Bad phosphorylation, could orchestrate the decision to undergo endothelial apoptosis by mitochondria permeabilization, cytochrome *c* release and further activation of the caspases proteolytic cascade (Figure 7). Undoubtedly, more experimental efforts will be required to elucidate this issue.

## 3. Experimental Section

### 3.1. Cell Culture

Bovine aortal endothelial (BAE) cells were grown in Dulbecco’s Modified Eagle Medium (DMEM) containing 1 g/L glucose, 10% fetal calf serum, 2 mM glutamine, 50 U/mL penicillin, 50 µg/mL streptomycin, 1.25 µg/mL amphotericin B. Human umbilical vein endothelial (HUVE) cells were isolated from umbilical cords as previously described [29] and grown on gelatin-coated dishes in Medium 199 containing 10 mM HEPES, 20% fetal calf serum, 2 mM glutamine, 100 µg/mL heparin and 30 µg/mL endothelial cell growth supplement (ECGS, Sigma-Aldrich, St Louis, MI, USA), 50 U/mL penicillin, 50 µg/mL streptomycin, 1.25 µg/mL amphotericin B. Passages three to seven were used for experiments. Human colon carcinoma cell line HCT-116 (kind gift from Prof. B. Vogelstein, Johns Hopkins Kimmel Comprehensive Cancer Center) and human fibrosarcoma HT-1080 were grown in DMEM containing 4.5 g/L glucose, 10% fetal calf serum, 2 mM glutamine, 50 U/mL penicillin, 50 µg/mL streptomycin, 1.25 µg/mL amphotericin B. All cell lines were maintained at 37 °C and humidified 5% CO_2_ atmosphere. All cell culture reagents were purchased from Gibco BRL (Grand Island, NY, USA). 

### 3.2. Antibodies

Mouse anti-PARP antibody (BD Biosciences) recognizes the 116 kDa full length form of PARP and the 85 kDa cleavage fragment. Mouse anti-cleaved-Lamin A antibody (Cell Signaling Technology) detects the small cleavage fragment of lamin A (28 kDa). Anti-Bad and anti-phospho-Bad (Ser 112) rabbit polyclonal antibodies were purchased from Cell Signaling Technology. Rabbit anti-GAPDH antibody was purchased from Nordic BioSite. Mouse anti-cytochrome *c* antibody was from BD Biosciences. Goat anti-rabbit and anti-mouse secondary HRP-conjugated antibodies were from PIERCE.

### 3.3. Treatments

Aeroplysinin-1 was dissolved in DMSO and stored at −20 °C. Cells were grown until 75% confluence and treated for 14 h with aeroplysinin-1 or vehicle (DMSO). For transduction pathway studies, cells were starved overnight by serum deprivation and treated for 2 h with aeroplysinin-1. Then, cells were stimulated for 10 min with medium containing 10% FBS and harvested for analysis. In some experiments, either the pan inhibitor of caspases z-VAD-fmk at 25 μM or the caspase-9 selective inhibitor z-LEHD-fmk at 20 μM was used (Enzyme Systems Products, Livermore, CA, USA). Cells were pre-treated with the caspase inhibitor for 30 min prior to treatment with aeroplysinin-1.

### 3.4. Cell Growth Assays

For cell growth curve, 5 × 10^4^ cells in a total volume of 500 µL of their respective complete growth medium were incubated (37 °C, 5% CO_2_ in a humid atmosphere) in each well of 24-well plates with 0, 1, 3, 5 or 10 µM of aeroplysinin-1. After 24 and 48 h, medium was removed, cells were washed twice with PBS, and the cell monolayers were detached with 500 µL of trypsin. The cellular suspension was diluted with 9.5 mL of Isoton diluent and counted by using a Coulter counter. Each concentration was tested in quadruplicate. For 3-(4,5-dimethylthiazol-2-yl)-2,5-diphenyltetrazolium bromide (MTT; Sigma Chemical Co., St. Louis, MO) dye reduction assay (MTT), 96-well microplates were used. 3 × 10^3^ cells in a total volume of 100 µL of complete medium were incubated in each well with serial dilutions of aeroplysinin-1. After 3 days of incubation in the dark (37 °C, 5% CO_2_ in a humid atmosphere), 10 µL of MTT (5 mg/mL in PBS) was added to each well and the plate was incubated for a further 4 h (37 °C). The resulting formazan was dissolved in 150 µL of 0.04 N HCl-2 propanol and read at 550 nm. All determinations were carried out in triplicate. IC50 values were calculated as those concentrations of compound yielding 50% cell survival, taking the values obtained for control as 100%.

### 3.5. Hoechst Staining

Cells plated on coverslips and grown to 75% confluence were treated during 14 h with aeroplysinin-1 at 3 and 10 µM. Then, the cells were fixed with formalin solution (Sigma), washed with phosphate-buffered saline (PBS) and stained with 1 µg/mL Hoechst in PBS. Coverslips were mounted on slides using Dakocytomation Fluorescent Mounting Medium (Dako) and observed under a fluorescence microscope (Leica, TCS-NT).

### 3.6. Cell Cycle Analysis

After treatment, attached and detached cells were harvested and centrifuged. Pellets were washed with PBS and suspended in 250 µL of ice-cold PBS. For fixation, 70% ice-cold ethanol was added while continuous gentle vortexing and samples were kept on ice for 1 h. Finally, cells were centrifuged and washed twice with PBS, suspended in 1 mL propidium iodide staining solution (40 µg/mL propidium iodide and 0.1 mg/mL RNase-A in PBS) and incubated for 1 h at 37 °C protected from light. The percentage of subG1 cells was determined using a MoFlo Dakocytomation flow cytometer (Beckman Coulter).

### 3.7. Flow Cytometry Analysis after Anexin V and 7-Aminoactinomycin D Staining

Apoptosis was examined by flow cytometry with the Annexin V-PE apoptosis kit (Pharmingen, BD Biosciences, San Agustin de Guadalix, Spain). BAE cells were incubated in the absence or presence of aeroplysinin-1 in complete growth medium. After 14 h of incubation, cells were washed and stained with phycoerythrin (PE)-labeled Annexin V and 7-aminoactinomycin D (7AAD), following manufacturer's instructions. Samples were analyzed by using a MoFlo Dakocytomation flow cytometer (Beckman Coulter), and the 7AAD−/PE-Annexin V−, 7AAD−/PE-Annexin V+, 7AAD+/PE-Annexin V+ and 7AAD+/PE-Annexin V− populations, corresponding to viable (Q3), early apoptotic (Q4), late apoptotic (Q2) and necrotic (Q1) cells respectively, were evaluated.

### 3.8. Western-Blot

Cells were lysed in Laemmli’s loading buffer 2× and boiled for 5 min at 95 °C. Samples were separated by SDS-PAG electrophoresis and blotted onto nitrocellulose membranes using standard procedures. After blocking in TBS-T plus 5% non-fat dry milk, membranes were probed with primary antibodies overnight at 4 °C. Then, the membranes were washed in TBS-T and probed with horseradish peroxidase (HRP)-conjugated secondary antibodies in a blocking solution for 1 h at room temperature. After washing, membranes were developed using the ECL™ system (Amersham Biosciences). For antibody re-probing, membranes were incubated in stripping solution (62.5 mM Tris-HCl pH 6.8, 2% SDS and 0.77% β-mercaptoethanol) for 30 min at 50 °C with shaking.

### 3.9. Caspase Activity Assay

After treatments, attached and detached cells were harvested. A total of 5 × 10^5^ cells were suspended in 25 μL PBS and snap-frozen in wells of a 96-well opaque microtiter plate on liquid nitrogen. After thawing on ice, 50 μL of assay buffer [13] containing 50 μM of fluorogenic substrate (Peptide Institute, Osaka, Japan) was added to each well. Caspase-3 and -8 assay buffer: 100 mM HEPES pH 7.2, 10% sucrose, 0.1% CHAPS; caspase-2 and -9 assay buffer: 0.1% MES pH 6.5, 10% poly-ethylene glycol, 0.1% CHAPS. Caspase-2, -3, -8 and -9 selective substrates Ac-VDVAD-AMC, Ac-DEVD-AMC, Ac-IETD-AMC and Ac-LEHD-AMC respectively, were used. Cleavage of the fluorogenic substrates was monitored for 30 min at 37 °C using a Fluoroscan II microplate reader (Thermo Electron Co., Waltham, MA, USA) using 355/460 nm excitation/emission wavelengths. Slopes of the fluorescence curves obtained were used as activity values and were represented as percentage of control values. Data from duplicate samples were used in each experiment.

### 3.10. Detection of Cytochrome c Release in Permeabilized Cells

Attached and detached BAE cells treated with aeroplysinin-1 for 14 h were harvested. After washing with PBS, 2 × 10^6^ cells were suspended in 100 μL of assay buffer (MSH buffer supplemented with 50 mM KCl, 1 mM EGTA, 5 mM succinate, 5 mM MgCl_2_), permeabilized with 10 μg digitonin for 5 min at room temperature and centrifuged at 13,000 rpm for 5 min. The supernatants containing soluble cytosolic fraction as well as the pellets containing mitochondria were mixed with Laemmli’s buffer. Finally, the samples were boiled for 5 min at 95 °C and cytochrome *c* release was analyzed by Western-blot.

### 3.11. Statistical Analysis

All data are expressed as means ± standard deviation (SD). One-tailed Student’s *t*-test was used to compare the actual difference between two means. *p* < 0.05 was considered to be statistically significant.

## 4. Conclusions

In summary, the data here show that the brominated compound aeroplysinin-1, isolated from a marine sponge, is able to induce apoptosis in endothelial cells in a pathway involving Bad dephosphorylation, mitochondrial cytochrome *c *release and activation of caspases. In addition to the previously reported capability of aeroplysinin-1 to interfere with several key steps of angiogenesis *in vitro*, this apoptogenic activity could contribute to the observed *in vivo* antiangiogenic activity of this compound.
